# Influence of Floral Strip Width on Spider and Carabid Beetle Communities in Maize Fields

**DOI:** 10.3390/insects15120993

**Published:** 2024-12-15

**Authors:** Jia-Lu Li, Lan-Mei Huang, Zi-Yi Xiang, Jian-Ning Zhao, Dian-Lin Yang, Hui Wang, Yan-Jun Zhang

**Affiliations:** 1 Agro-Environmental Protection Institute, Ministry of Agriculture and Rural Affairs, No. 31, Fukang Road, Nankai District, Tianjin 300191, China; 2 School of Resources and Environment, Northeast Agricultural University, Harbin 150006, China

**Keywords:** staple crops, natural pest control, natural enemies, biodiversity conservation, floral strips, biodiversity enhancement, conservation agriculture, maize fields, strip width, agroecosystem management

## Abstract

The study explored the impact of floral strip width on the spider and carabid beetle communities in maize fields over two years. Three widths of floral strips (2 m, 4 m, and 6 m) were compared with maize-only control strips to evaluate species diversity and distribution. The results showed significant differences in both spider and carabid populations between floral and control strips, with 4 m and 6 m widths consistently harboring higher biodiversity. The results also showed distinct community clustering within floral strips in 2021, which became more cohesive by 2022. Further analysis validated significant community dissimilarities between different strip widths and controls, highlighting the ecological advantages of wider floral strips for enhancing natural enemy biodiversity. Spider activity density was notably higher in floral strips than in adjacent farmland, peaking at the edges of 4 m-wide strips and decreasing in 6 m-wide strips, with the lowest density in 2 m-wide strips. Carabid beetle activity density varied considerably with strip width and proximity to the edge, typically peaking at the edges of wider strips. Spiders were more responsive to strip width than carabid beetles. Based on these findings, we suggest using 4 m- or 6 m-wide floral strips to enhance biodiversity and natural pest control in agricultural landscapes; the floral strips narrower than 4 m (such as 2 m) could not support optimal biodiversity, as spiders and carabid beetles do not disperse far into the maize field, with spiders having dispersal distances of less than 3 m and carabid beetles less than 10 m. Vegetation characteristics significantly influenced spider and carabid communities, impacting species richness, diversity indices, and community structures across two study years. These insights highlight the necessity of thoughtfully designing floral strips to enhance biodiversity and natural pest control in agricultural landscapes.

## Simple Summary

This study investigated how the width of flower strips next to maize fields influences the diversity of spiders and ground beetles, which are important natural enemies. Over two years, flower strips of three different widths—2 m, 4 m, and 6 m—were compared to maize-only areas. The results showed that wider flower strips (4 m and 6 m) supported more diverse and abundant communities of spiders and beetles compared to narrower strips and maize-only areas. Spiders were more active near the edges of the strips, with the highest activity in the 4 m strips, while beetles showed varying activity based on width and distance from the edge. The study also found that vegetation characteristics played a key role in shaping these communities. These findings emphasize the importance of designing wider flower strips to boost biodiversity and support natural pest control in farmland, benefiting sustainable agriculture and ecosystem health.

## 1. Introduction

Farmland biodiversity encompasses the species inhabiting agricultural landscapes and the intricate trophic relationships among them [1]. Its rich genetic and species diversity is crucial for sustainable agricultural production [2]. Modern agricultural practices, such as expanding agricultural areas and reducing crop diversity to facilitate mechanization and higher yields, have simplified agricultural landscapes and intensified environmental impact [3], thereby adversely affecting farmland biodiversity [4]. To mitigate these negative impacts, various conservation measures have been implemented in recent decades [5]. Field boundaries, crucial non-crop habitats within agricultural ecosystems, play a significant role in agricultural landscapes [6]. Floral strips—specifically designed vegetated areas along field boundaries featuring diverse plantings such as grasses, legumes, and composite plants—serve as managed components of agricultural ecosystems. These strips provide a variety of ecological services, including biodiversity conservation, climate change mitigation, and esthetic and recreational values. Importantly, floral strips can support the population and efficacy of beneficial arthropods, particularly predators and pollinators, which play key roles in pest control and crop pollination in agriculture [7,8,9,10,11]. Wider floral strips exhibit higher plant diversity, and larger areas support more diverse vegetation and community compositions, aligning with the “species-area” hypothesis [12]. Studies have demonstrated that the diversity of plant species within these conservation areas influences the effectiveness of pest control, with species-rich floral strips attracting a wider range of beneficial arthropods, such as carabid beetles and parasitoids, which contribute to pest regulation [13,14]. Reserving some fallow fields facilitates species migration and colonization, thereby reducing the risk of biodiversity loss [15]. Well-designed agricultural landscapes can effectively mitigate biodiversity decline caused by intensive agricultural practices and environmental pollution [5,16,17], enhancing the spatial and temporal distribution patterns of natural enemies crucial for pest control.

Biological pest control heavily relies on diverse natural enemies, with even small non-crop habitats playing a crucial role in maintaining this diversity [17]. Increasing the diversity of natural enemies through landscape-level approaches enhances their effectiveness in controlling pests [18]. Diverse natural enemy communities influence pest behavior, physiology, morphology, and life history, thereby shaping pest community dynamics and habitat preferences [19]. Throughout the crop growing season, natural enemies migrate from non-crop habitats into adjacent fields, augmenting the ratio of natural enemies to prey and improving pest control efficiency [20]. Even minimal predation by natural enemies can significantly suppress pest populations through what is known as the enemy risk effect [20]. Therefore, assessing natural enemy diversity is essential for evaluating the effectiveness of biological pest control. Non-crop habitats, such as floral strips at farmland boundaries, are critical for conserving natural enemies and enhancing biological pest control within agricultural landscapes, influencing spatial distribution and temporal dynamics [21]. However, variations in habitat configuration, area, vegetation type, and structure can significantly affect the composition of pest insects and natural enemy communities within these habitats [22,23].

Research emphasizes the role of non-crop habitats in conserving biodiversity and enhancing integrated pest management strategies [6,16,24]. These studies provide theoretical insights into how non-crop habitats and vegetation influence natural enemy communities, including spiders and carabids, which play crucial roles in regulating pest populations [25,26]. Changes in landscape structure can lead to significant alterations in carabid and spider communities within agricultural landscapes [27]. Secondary vegetation succession on abandoned agricultural land also influences carabid community development across various trophic levels [28]. Floral strips have been established as beneficial habitats in various agricultural systems. Prior studies have demonstrated their effectiveness in crops like wheat, barley, potatoes, and beans [21,29,30]. However, the application of floral strips in maize fields remains unexplored. This study aims to provide clear recommendations for optimizing the width of conservation strips to enhance beneficial arthropod conservation, ultimately contributing to sustainable maize pest management. Specifically, the study will demonstrate how different widths of floral strips influence the diversity and activity of spiders and carabid beetles. By identifying the optimal strip width for maximizing arthropod conservation, our study will offer practical guidance for designing conservation strips that support natural pest control in maize fields. Understanding the dynamics between the width of floral strips and natural enemies in maize fields is crucial for identifying how floral strips can support beneficial species. The findings of this study will provide practical recommendations for optimizing floral strip design in maize cultivation, ultimately enhancing sustainable maize pest management strategies.

## 2. Materials and Methods

### 2.1. Study Area

The experimental study was conducted at Songfang Farm, Qihe County, Dezhou City, Shandong Province, China (36°64′34″ N, 116°58′58″ E). This region has a warm-temperate semi-humid monsoon climate with distinct hot and cold seasons, as well as wet and dry periods. The elevation is approximately 20 m above sea level, with an average annual temperature of 13.4 °C and annual precipitation of 622 mm. The frost-free period spans 217 days (from mid-April to late-November). The soil is primarily alluvial, with a pH value of 7.93. Soil characteristics include 9.47 g·kg^−1^ organic matter, 4.48 mg·kg^−1^ ammonium nitrogen, 15.15 mg·kg^−1^ nitrate nitrogen, 1.44 g·kg^−1^ total nitrogen, and 16.10 mg·kg^−1^ available phosphorus. The local cropping system predominantly involves a winter wheat–summer maize rotation.

### 2.2. Experiment Design

**Figure 1:**
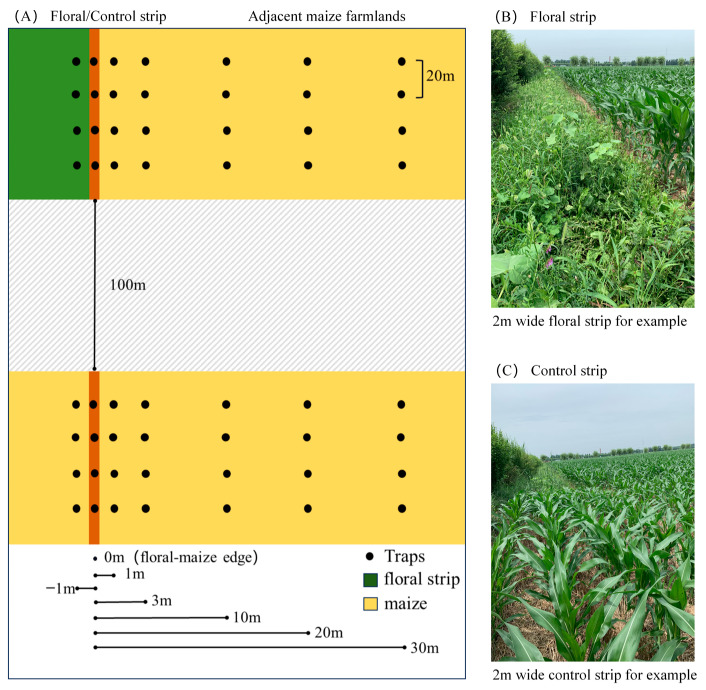
(**A**) schematic representation showing the spatial arrangement of floral/control strips and their arthropod sampling sites within each replicate, (**B**) a photograph of 2 m-wide floral strip as example, and (**C**) a photograph of 2 m-wide maize planted control strip as example.

In October 2019, twelve fields were selected, each at least 200 m apart. Four fields were randomly chosen to serve as replicates for each width category. Each field had a north–south orientation, measuring 300 m in length and 150 m in width. The fields were divided into three equal sections along the long side. A floral strip was planted along the boundary of the first section, while a control strip, which involved planting maize, was situated along the boundary of the third section (Figure 1A). Both the floral and control strips were 100 m long and spaced more than 100 m apart, with widths of 2 m, 4 m, and 6 m. The floral strips were constructed by sowing a seed mixture consisting of 65% perennial ryegrass *Lolium perenne* L. (Poaceae) (*w*/*w*), 5% alfalfa *Medicago sativa* L. (Fabaceae) (*w*/*w*), 5% vetch *Vicia sepium* L. (Fabaceae) (*w*/*w*), 5% white clover *Trifolium repens* L. (*w*/*w*), 5% red clover *Trifolium pratense* L. (Fabaceae) (*w*/*w*), 5% chicory *Cichorium intybus* L. (Asteraceae) (*w*/*w*), 5% garden cosmos *Cosmos bipinnatus* Cav. (Asteraceae) (*w*/*w*), and 5% sage *Salvia japonica* Thunb. (Poaceae) (*w*/*w*). The strips were managed extensively with no fertilizers or agrochemicals and were mowed 2–3 times annually, depending on growth conditions. Floral strips with widths of 2 m (2m-T), 4 m (4m-T), and 6 m (6m-T) were established alongside maize-planted control strips (2m-C, 4m-C, and 6m-C). A pair of 2 m-wide floral and control strips is illustrated in Figure 1B,C. During the 2021 and 2022 growing seasons, investigations and arthropod sampling were conducted within both the floral and control strips, as well as in the adjacent maize fields. Surface pitfall traps were deployed during the maize tasseling and silking stages to sample spiders and carabids [31]. Pitfall traps were arranged in four parallel sampling locations spaced 20 m apart, each representing a distinct sampling point within the study area. These were placed along the centerline of the floral strips and their corresponding control strips to collect surface-dwelling arthropods (Figure 1A). To assess the influence of floral strip width and vegetation diversity on spider and carabid communities within maize fields, traps were positioned at the floral-maize edge (0 m), within the floral strip (−1 m), and at distances of 1 m, 3 m, 10 m, 20 m, and 30 m from the edge of the maize fields.

### 2.3. Vegetation Survey

During the experiment, we used the sampling method to investigate and record the names of plant species, along with their numbers and coverage of the floral strips, while simultaneously capturing insects [32,33]. Subsequently, vegetation diversity indices—such as the Importance Value, Shannon Diversity Index, and Pielou’s Evenness Index—were calculated using this survey data to evaluate the ecological impacts of the treatments [34].

### 2.4. Arthropod Sampling and Identification

Pitfall traps were constructed from plastic cups measuring 7.8 cm in diameter and 10 cm in depth, filled with one-third saturated saltwater and a few drops of detergent. Positioned with the rim at ground level, the traps were left in place for 48 h [31,35,36]. Arthropod samples collected from four parallel traps were pooled into a single sample per replicate, preserved in plastic bottles containing 75% ethanol, and transported to the laboratory for further analysis. Spider families were categorized into hunting and web-building spiders based on their predatory behaviors [37], while carabid beetles were classified into carnivorous and omnivorous groups according to their feeding preferences [38]. Spider identification was conducted up to the family level, whereas carabid beetles were identified at the species level, with certain specimens verified by experts from Hebei University and Beijing Forestry University.

### 2.5. Data Analysis

Species richness, active density, and the Shannon Diversity Index of spiders and carabid beetles in the 2m-T, 4m-T, 6m-T, 2m-C, 4m-C, and 6m-C were compared using analysis of variance (ANOVA). Community structure similarities between spiders and carabid beetles across these groups were assessed via non-metric multi-dimensional scaling (NMDS), based on the chord-normalized expected species shared (CNESS) similarity coefficient, with differences tested using analysis of similarities (ANOSIM). The spillover effects of spiders and carabid beetles were analyzed by examining active density at various distances from the strip edge within maize fields. Spearman correlation was used to evaluate the relationships between spider or carabid beetle active density and distances from the strip edge. Redundancy analysis (RDA) assessed the influence of floral strips on the community structure and distribution of these arthropods. Statistical analyses, including ANOVA and general linear regression, were performed using SAS 9.4 [39]. Multiple comparison methods were applied using Duncan’s new multiple range test. NMDS, ANOSIM, and Spearman correlation analyses were conducted using R 4.0.2 [40], while RDA was performed using CANOCO 5 [41].

## 3. Results

### 3.1. Differences in Composition and Structure of Natural Enemy Communities Between Floral Strips and Control Strips

A total of 1736 spiders from 12 families were collected using surface traps during 2021–2022 (Table 1). Wolf spiders (Lycosidae) predominated, comprising 71.49% of the total individuals. Other common families included Nesticidae (8.70%), Dictynidae (8.12%), and Linyphiidae (7.03%). Hunting spiders constituted 75.40% of the total, represented by eight families, while web-building spiders constituted 24.60%, represented by four families. The number of spider families (*F* = 9.8, df = 7, *p* = 0.035) and individuals (*F* = 14.88, df = 7, *p* = 0.018) in floral strips was significantly higher compared to control strips. A total of 285 carabid beetles were collected using surface traps, representing 19 species. Dominant species included *Chlaenius micans* (20.00%) and *Calosoma lugens* (13.68%). Carnivorous and omnivorous species constituted 54.74% and 45.26% of total carabids, respectively. Although not statistically significant, the number of carabid beetle species (*F* = 2.306, df = 7, *p* = 0.204) and individuals (*F* = 3.298, df = 7, *p* = 0.144) tended to be higher in floral strips compared to control strips.

**Figure 2:**
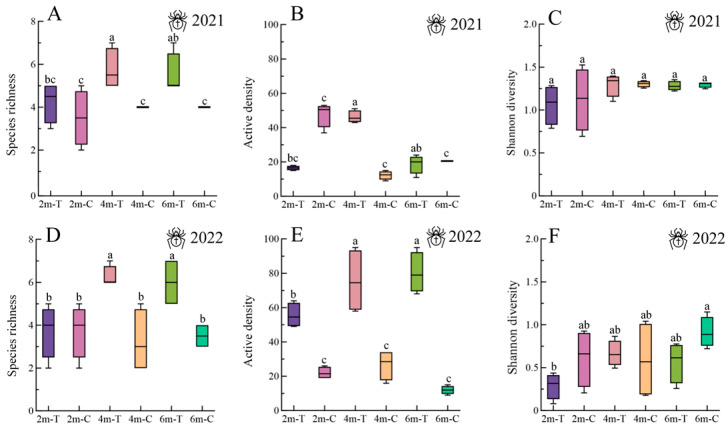
Species richness, active density, and Shannon Diversity Index of spiders in floral strips and control strips. (**A**–**C**) in 2021 and (**D**–**F**) in 2022. 2m-T: 2m-wide floral strip, 2m-C: 2 m-wide control strip. 4m-T: 4 m-wide floral strip, 4m-C: 4 m-wide control strip. 6m-T: 6 m-wide floral strip, 6m-C: 6 m-wide control strip. Boxplots display the interquartile range (25–75%; box) and the median (line in the box). Whiskers represent 1.5 times the lower or upper interquartile range. Different lowercase letters above bars indicate significant differences among treatments.

**Figure 3:**
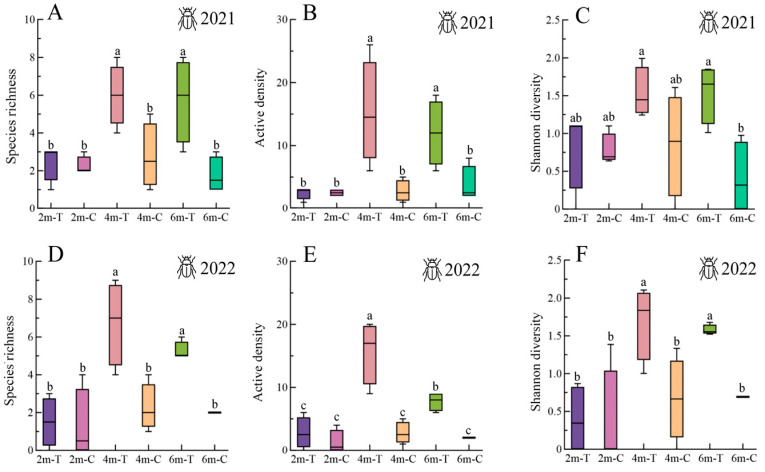
Species richness, active density, and Shannon Diversity Index of carabids in floral strips and control strips. (**A**–**C**) in 2021 and (**D**–**F**) in 2022. 2m-T: 2 m-wide floral strip, 2m-C: 2 m-wide control strip. 4m-T: 4 m-wide floral strip, 4m-C: 4 m-wide control strip. 6m-T: 6 m-wide floral strip, 6m-C: 6 m-wide control strip. Boxplots display the interquartile range (25–75%; box) and the median (line in the box). Whiskers represent 1.5 times the lower or upper interquartile range. Different lowercase letters above bars indicate significant differences among treatments.

The ANOVA results revealed significant differences in spider populations between 2021 (species richness: *F* = 4.4, df = 23, *p* = 0.009; active density: *F* = 57.01, df = 23, *p* < 0.001) (Figure 2A,B) and 2022 (species richness: *F* = 5.86, df = 23, *p* = 0.002; active density: *F* = 31.72, df = 23, *p* < 0.001) (Figure 2D,E). Specifically, both species richness and active density were notably higher in the 4 m-wide floral strips compared to the 2 m-wide strips in both years. In 2022, the 6 m-wide floral strips also exhibited significantly higher species richness and active density (Figure 2D,E). Furthermore, the 4 m- and 6 m-wide floral strips consistently showed higher species richness (2021: *F* = 4.4, df = 23, *p* = 0.009; 2022: *F* = 5.86, df = 23, *p* = 0.002) (Figure 2A,B) and active density (2021: *F* = 57.01, df = 23, *p* < 0.001; 2022: *F* = 31.72, df = 23, *p* < 0.001) (Figure 2D,E) compared to their respective control strips. For carabids, significant differences were observed in species richness (2021: *F* = 6.63, df = 23, *p* = 0.001; 2022: *F* = 10.33, df = 23, *p* < 0.001), active density (2021: *F* = 7.21, df = 23, *p* < 0.001; 2022: *F* = 18.69, df = 23, *p* < 0.001), and Shannon Diversity Index (2021: *F* = 3, df = 23, *p* = 0.016; 2022: *F* = 6.89, df = 23, *p* < 0.001) between the 4 m- and 6 m-wide floral strips compared to the 2 m-wide strips (Figure 3A–F). Additionally, the 4 m- and 6 m-wide floral strips consistently exhibited a higher species richness, active density, and Shannon Diversity Index compared to their respective control strips (Figure 3A–F).

**Figure 4:**
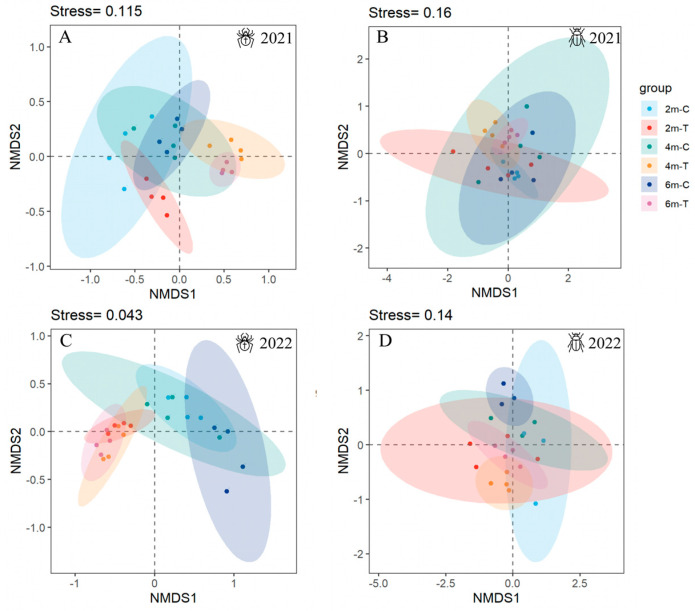
Non-linear multi-dimensional scaling (NMDS) based on chord measure of spiders and carabids communities in floral strips and control strips. (**A**) Spiders in 2021, (**B**) carabids in 2021, (**C**) spiders in 2022, and (**D**) carabids in 2022.

The NMDS results indicated that spider communities in the 4 m- and 6 m-wide floral strips were highly clustered in 2021, distinctly separated along both NMDS1 and NMDS2 from the communities in the 2 m-wide floral strips (Figure 4A). In contrast, by 2022, there was no clear separation observed among the spider communities in the 2 m-, 4 m-, and 6 m-wide floral strips (Figure 4B). However, spider communities in these floral strips did show separation from their respective control strips (Figure 4A,C). The ANOSIM results confirmed that in 2021 the spider communities in the 2 m-, 4 m-, and 6 m-wide floral strips were significantly different from each other, whereas this distinction was not observed in 2022 (Table 2). Additionally, significant differences were observed between each floral strip and their respective control strips in both 2021 and 2022 (Table 2). Similarly, the carabid communities in the 4 m- and 6 m-wide floral strips were highly clustered in 2021, distinctly separated along NMDS2 from the communities in the 2 m-wide floral strips (Figure 4B). In 2022, a clear separation was observed only between the spider communities in the 4 m- and 6 m-wide floral strips (Figure 4D). Carabid communities in the 4 m- and 6 m-wide floral strips also showed separation from their respective control strips (Figure 4B,D). ANOSIM results confirmed that the carabid communities in the 4 m- and 6 m-wide floral strips were significantly different from those in the 2m-wide floral strips in 2021, and the carabid communities in the 4 m-wide floral strips were significantly different from those in the 6 m-wide floral strips in 2022 (Table 2). Additionally, compared to their respective control strips, the 4 m-wide floral strips were distinctly separated in both years, while the 6 m-wide floral strips were only separated in 2022 (Table 2).

### 3.2. Influence of Floral Strips on the Spatial Distribution of Spiders and Carabid Beetles

**Figure 5:**
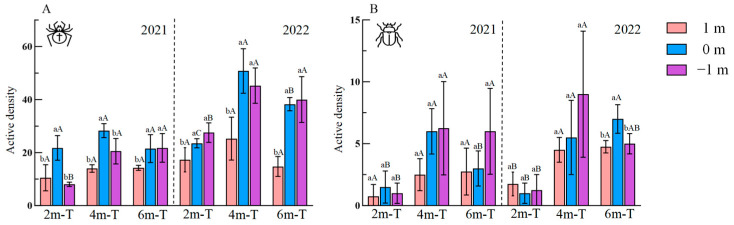
Active density of (**A**) Spiders and (**B**) carabids within floral strip (−1 m), at strip edge (0 m), and in adjacent farmland (1 m). 2m-T: 2 m-wide floral strip, 4m-T: 4 m-wide floral strip, 6m-T: 6 m-wide floral strip. Data presented as mean ± SE. Different lowercase and uppercase letters above bars indicated significant differences among distances for each width of floral strip and widths for each distance from edge, respectively.

The width of floral strips (2021: *F* = 12.15, df = 11, *p* < 0.001; 2022: *F* = 26.97, df = 11, *p* < 0.001) and their distance from the strip edge (2021: *F* = 23.26, df = 11, *p* < 0.001; 2022: *F* = 39.20, df = 11, *p* < 0.001) significantly influenced spider active density over two years. Spider active density at the strip edge was notably higher within floral strips compared to adjacent farmland in both 2021 (2m-T: *F* = 13.81, df = 11, *p* = 0.002; 4m-T: *F* = 10.75, df = 11, *p* = 0.004; 6m-T: *F* = 6.82, df = 11, *p* = 0.016) and 2022 (2m-T: *F* = 8.52, df = 11, *p* = 0.008; 4m-T: *F* = 12.00, df = 11, *p* = 0.003; 6m-T: *F* = 24.90, df = 11, *p* < 0.001). Spider active density within the floral strips themselves was also significantly higher compared to adjacent farmland, except for the 2 m- and 4 m-wide floral strips in 2021 (Figure 5A). In terms of the distance from the strip edge, spider active density at the strip edge was significantly higher in the 4 m-wide floral strips compared to the 6 m-wide floral strips, which in turn was higher than the 2 m floral strips (*F* = 28.08, df = 11, *p* < 0.001) in 2022 (Figure 5A). Spider active density within the floral strips themselves was significantly higher in the 4 m- and 6 m-wide floral strips during both years (2021: *F* = 13.19, df = 11, *p* = 0.002; 2022: *F* = 7.52, df = 11, *p* = 0.012) (Figure 5A).

The width of floral strips significantly influenced carabid active density in both 2021 (*F* = 10.65, df = 11, *p* < 0.001) and 2022 (*F* = 18.96, df = 11, *p* < 0.001). Additionally, the distance from the strip edge also showed a significant effect on carabid active density in 2021 (*F* = 4.01, df = 11, *p* = 0.03). Specifically, regarding floral strip width, carabid active density at the strip edge of the 6 m-wide floral strip was significantly higher compared to within the floral strip itself and adjacent farmland in 2022 (2m-T: *F* = 8.52, df = 11, *p* = 0.008; 4m-T: *F* = 12.00, df = 11, *p* = 0.003; 6m-T: *F* = 24.90, df = 11, *p* < 0.001). Concerning the distance from the strip edge, carabid active density at the strip edge of the 4 m floral strips was significantly higher than at the 2 m- and 6 m-wide floral strips in 2021 (*F* = 9.00, df = 11, *p* = 0.007), and only higher than that at the 2 m-wide floral strips in 2022 (*F* = 10.63, df = 11, *p* = 0.004) (Figure 5B). Furthermore, carabid active density within the floral strips themselves was significantly higher in the 4 m- and 6 m-wide floral strips in 2021 (*F* = 2.31, df = 11, *p* = 0.037), and notably so in the 4 m-wide floral strips in 2022 (*F* = 15.34, df = 11, *p* = 0.001) (Figure 5B). Moreover, carabid active density in the farmland adjacent to the 4 m- and 6 m-wide floral strips was significantly higher compared to the 2 m-wide floral strips in 2022 (*F* = 10.63, df = 11, *p* = 0.019) (Figure 5B).

**Figure 6:**
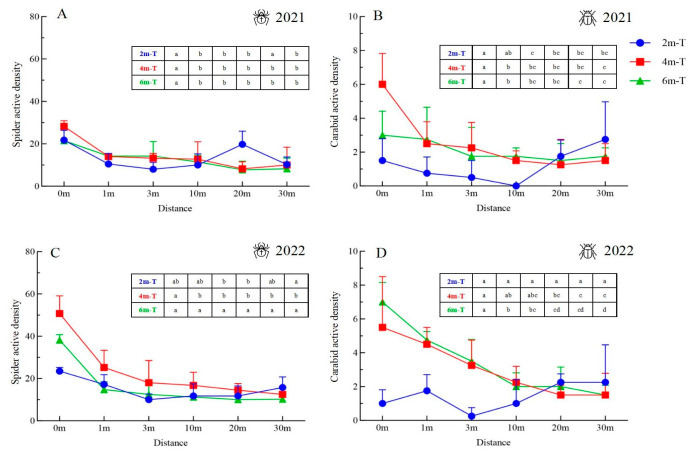
Active density of spiders and carabids at various distances from the strip edge into adjacent maize fields. (**A**) Spiders in 2021, (**B**) carabids in 2021, (**C**) spiders in 2022, and (**D**) carabids in 2022. 2m-T: 2 m-wide floral strip, 4m-T: 4 m-wide floral strip, 6m-T: 6 m-wide floral strip. Data presented as mean ± SE. Different lowercase in the tables denoted significant differences among distances for each width of floral strip.

Both spider and carabid active density in the farmlands adjacent to the 4 m- and 6 m-wide floral strips gradually declined with increasing distance from the strip edge. However, the active densities of spiders and carabids in the farmlands adjacent to the 2 m-wide floral strips initially declined and then increased (Figure 6). Spearman correlation analysis indicated a negative correlation between spider active density and distance from the strip edge, with significant differences observed in the farmlands adjacent to the 2 m, 4 m, and 6 m floral strips (Table 3). Similarly, there was a negative correlation between carabid active density and distance from the strip edge, with significant differences found in the farmlands adjacent to the 4 m and 6 m floral strips (Table 3). A positive but non-significant correlation was observed between carabid active density in the farmlands adjacent to the 2 m-wide floral strips and in the distance from the strip edge (Table 3). ANOVA results indicated significant differences in spider active density at various distances within the farmlands adjacent to the 2 m-, 4 m-, and 6 m-wide floral strips in 2021, which were more pronounced in 2022 (Table 3) (Figure 6A,B). For carabids, significant differences in active density were observed at various distances within the farmlands adjacent to the 4 m- and 6 m-wide floral strips (Table 3) (Figure 6C,D).

### 3.3. Impact of Floral Strip Vegetation Characteristics on Spider and Carabid Beetle Community Structure

In 2021, the floral strip supported a diverse flora, including four species each of Poaceae, Asteraceae, and Leguminosae, which constituted 28.45%, 27.75%, and 18.35% of the total plant population, respectively (Table 4). Additionally, there were two species of Malvaceae and one species each of Amaranthaceae, Campanulaceae, Chenopodiaceae, Convolvulaceae, Cucurbitaceae, Moraceae, and Solanaceae. By 2022, Asteraceae dominated the plant population at 39.76% with five species, followed by Poaceae at 31.19% (two species) and Leguminosae at 21.81% (three species). Malvaceae, Moraceae, and Rubiaceae each had 1 species. Dominant plants in the floral strips in 2021 included *Cichorium intybus*, *Setaria viridis*, *Digitaria sanguinalis*, and *Medicago sativa*. By 2022, dominant species were *Setaria viridis*, *Cichorium intybus*, *Medicago sativa*, *Cirsium arvense* var. *integrifolium*, and *Lolium perenne*, all with an Importance Value above 10%.

**Figure 7:**
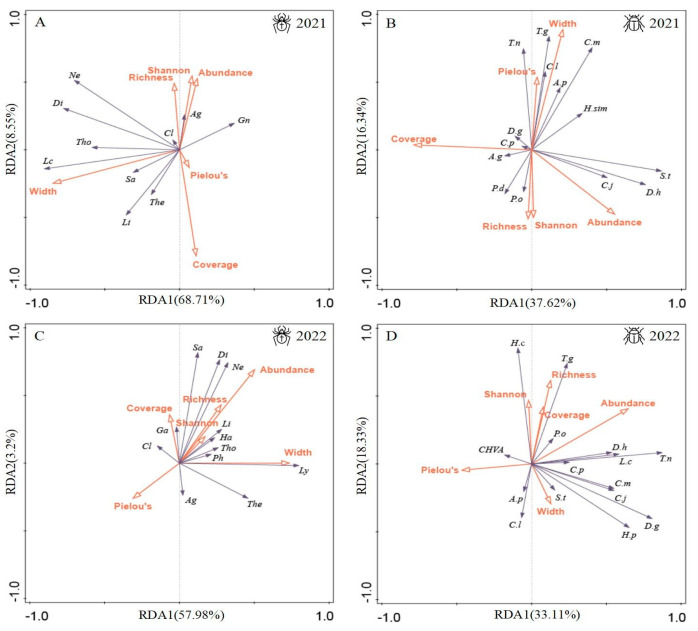
Redundance analysis (RDA) of spiders and carabids assemblage structures and vegetation characteristics of floral strips. (**A**) Spiders in 2021, (**B**) carabids in 2021, (**C**) spiders in 2022, and (**D**) carabids in 2022.

Floral strip width, vegetation coverage, abundance, richness, Shannon Diversity Index, and Pielou’s Evenness Index collectively explained 79.8% of the variation in spider community structure in 2021 and 63.8% in 2022, as well as 59.5% in 2021 and 69.5% in 2022 for carabid community structure (Table 5). Spider communities were significantly influenced by floral strip width in 2021 and marginally influenced in 2022, as well as by vegetation coverage in 2021 (Table 5). The RDA ordination plot indicated that the first two axes explained 77.26% of the variance in spiders and their environment in 2021 (Figure 7A) and 61.18% in 2022 (Figure 7C). Floral strip width showed prominently negative scores on the first axis in 2021 and positive scores in 2022, while vegetation coverage exhibited high negative scores on the second axis in 2021. The hunting spider family Lycosidae (Ly) correlated positively with floral strip width, whereas Agelenidae (Ag) showed a negative correlation with vegetation coverage (Figure 7A,C). Web-building spider families did not display significant correlations with vegetation characteristics. For carabids, communities were marginally influenced by floral strip width in 2021 and significantly influenced in 2022, while vegetation coverage significantly affected them only in 2021 (Table 5). The first two axes explained 53.96% in 2021 and 51.44% in 2022 of the variation in carabids and their environment (Figure 7B,D). Omnivorous species like *Amara plebeja* (A.p) and carnivorous species like *Calosoma lugens* (C.l) showed positive correlations with floral strip width in both 2021 and 2022 (Figure 7B,D), whereas omnivorous species *Scarites terricola* (S.t) and carnivorous species *Dolichus halensis* (D.h) exhibited negative correlations with vegetation coverage in 2021 (Figure 7B).

## 4. Discussion

### 4.1. Spider and Carabid Beetle Diversity in Floral Strips

Maintaining species diversity and community stability is crucial for enhancing overall ecosystem resilience [42]. Floral strips can create ecological niches by offering a diverse array of plant species and structural complexity, serving multiple roles as alternative hunting grounds, overwintering sites, and shelters for natural enemy arthropods, thereby enhancing spider and carabid diversity [29]. Our first hypothesis suggested that floral strips would support the greater diversity and active density of spiders and carabids compared to the control strips. This study confirmed that floral strips indeed harbored more spiders and carabids than control strips, supporting our initial hypothesis. Furthermore, the similarity of spider and carabid communities in floral strips of varying widths underscores the importance of strip design in supporting diverse and functional arthropod populations. It has been suggested that non-crop habitats like floral strips, by offering selective resources, can attract specific natural enemies more efficiently, thus helping to maintain balanced and resilient pest control communities [43,44,45]. This highlights the potential of floral strips to serve as a key component of integrated pest management strategies, enhancing the ecological resilience of agroecosystems [46]. The impact of floral strips on farmland biodiversity stems from the stability and structural complexity they provide. Artificially established floral strips can attract target natural enemies earlier and more selectively than control strips, thereby fostering resilient communities of these natural enemies [47].

### 4.2. Spatial Variation of Spiders and Carabid Beetles in Response to Floral Strip Width

Biodiversity dynamics within floral strips operate across spatial scales [48,49]. These non-crop habitats are pivotal in agricultural landscapes, profoundly shaping biodiversity distribution patterns. The size and configuration of floral strips directly impact farmland biodiversity [50,51]. Our second hypothesis posited that the active densities of spiders and carabids would increase with wider floral strips. The broader floral strips enhance non-agricultural habitat area, thereby providing increased food sources and larger habitats for natural enemies [52].

Furthermore, our hypothesis suggested that the active densities of spiders and carabids would decrease with distance from the floral strip within the farmland. The edge between crop and non-crop habitats is a dynamic zone of population interaction and a critical feature of agricultural landscapes [50,53,54,55]. Within this transition zone, ecosystems with distinct characteristics modify various system components and behaviors due to ecological differences and interactions [56]. This study found that spider active density at the strip edge was the highest and was significantly greater than in adjacent farmlands. In contrast, carabid active density showed a more gradual decline across distances, particularly in wider strips (4 m and 6 m), reflecting their greater adaptability and dispersal ability. These findings emphasize the critical role of strip width and composition in facilitating natural enemy dispersal [57]. For example, carnivorous carabids depend on floral strips for movement between habitats, feeding locations, and for evading agricultural disturbances [58]. Moreover, the spatial distribution of natural enemies involves species dispersing across distances, with populations from wider floral strips (4 m and 6 m) exhibiting more pronounced spillover effects into adjacent farmlands compared to narrower strips (2 m) [59]. The effective dispersal distance of natural enemy insects typically reaches up to 30 m in wheat farmlands, beyond which populations sharply decline [60]. Our study identified significant spillover effects of spiders and carabids into adjacent maize farmland from 4 m- to 6 m-wide floral strips. However, food sources and shelters within floral strips may attract spiders and carabids, prompting migration to these areas [61,62]. In maize fields, the effective dispersal distance was approximately 30 m in our study, indicating the intrinsic and fixed dispersal capacities of spiders and carabids regardless of edge type [60]. Carabids surviving in intensively managed and fragmented farmland tend to be widely distributed species with strong adaptability [63], migrating to non-crop habitats within farmlands during adverse conditions.

### 4.3. Correlation Between Vegetation Characteristics and Spider and Carabid Beetle Communities

Certain vegetation types play a pivotal role in supporting diverse natural enemy populations [28]. Thus, our third hypothesis posited that the vegetation characteristics of floral strips would influence the diversity and community traits of spiders and carabids. Redundancy analysis integrating spider and carabid communities, floral strip width, and vegetation characteristics revealed significant impacts of strip width and vegetation coverage on community structure [64,65]. The hunting spider family Lycosidae exhibited a positive correlation with floral strip width, while the hunting spider family Agelenidae showed a negative correlation with vegetation coverage [66]. In contrast, web-building families did not demonstrate significant correlations with vegetation characteristics, and it may only increase the abundance of potential prey [67]. These findings suggest varied responses among spider groups to environmental factors, with hunting families thriving in larger habitats with sparser vegetation, whereas web-building families exhibit either less preference or greater adaptability to habitat conditions [26,68]. Regarding carabids, the omnivorous species *A. plebeja* and the carnivorous species *C. lugens* showed positive correlations with floral strip width, while the omnivorous species *S. terricola* and the carnivorous species *D. halensis* exhibited negative correlations with vegetation coverage. These findings suggest that specific carabid species respond differently to floral strip characteristics, with some benefiting from wider strips and others being more sensitive to vegetation coverage. This underscores the importance of tailoring conservation strategies to accommodate the varying ecological requirements of carabid species. This result underscores the resilience of carabids surviving in intensively managed and fragmented farmland, which often display strong adaptability [63]. While vegetation species richness and structural heterogeneity are critical factors influencing carabid communities [69], no significant correlations were observed in this study between spider and carabid communities and variables such as vegetation species richness, abundance, Shannon Diversity Index, and Pielou’s Evenness Index. Furthermore, seasonal changes in vegetation phenology and arthropod life histories contribute to turnover in community composition [70]. Over the two-year study period, fluctuations in vegetation within floral strips were noted; for example, populations of Poaceae declined while Asteraceae and Leguminosae increased. Consequently, spider active density and Shannon diversity were higher in 2022 compared to 2021. These observations align with prior research documenting biodiversity responses to landscape structure changes and vegetation succession processes [28,69,71]. However, the two-year duration of our study may be insufficient to fully capture changes in spider and carabid communities in response to vegetation succession over longer temporal scales. Therefore, future research should encompass long-term investigations.

## 5. Conclusions

Our study highlights the critical role of floral strips in enhancing arthropod diversity and active densities in agricultural landscapes. Wider floral strips and their proximity to farmland were positively associated with spider and carabid densities, while vegetation characteristics within strips significantly influenced arthropod community traits. These findings underscore the importance of designing tailored conservation strategies to support beneficial arthropods and promote sustainable pest management. Understanding these dynamics is crucial for optimizing the effectiveness of floral strips as tools for conserving arthropod biodiversity and reducing the reliance on chemical pesticides.

Moreover, while our study focused on the role of floral strips in supporting natural enemy populations, we recognize that their potential impact on pest suppression in crops like maize remains an important area for future research. Therefore, future studies should assess how floral strip width influences pest populations in crops, both in terms of direct effects (e.g., pest mortality) and indirect effects (e.g., the presence of natural enemies). Experimental designs incorporating pest monitoring alongside evaluations of natural enemy richness and activity would provide valuable insights into the role of floral strips in enhancing biological pest control. By exploring these temporal and long-term effects, future research can further refine conservation strategies and maximize the ecological benefits of floral strips in agricultural landscapes.

## Figures and Tables

**Table 1:** Spider and carabid community composition in floral strips and control strips.

Groups	Types	Codes	Families/Species	Floral Strips		Control Strips	
				2021	2022	2021	2022
Spiders	Hunting	Ag	Agelenidae	6	16	0	2
		Cl	Clubionidae	2	2	0	0
		Gn	Gnaphosidae	4	6	2	5
		Ly	Lycosidae	237	740	71	193
		Ph	Pholcidae	0	1	0	0
		Sa	Salticidae	2	4	0	0
		The	Theridiidae	4	5	0	0
		Tho	Thomisidae	5	1	0	1
	Web-building	Di	Dictynidae	46	31	45	19
		Ha	Hahniidae	0	13	0	0
		Li	Linyphiidae	57	10	44	11
		Ne	Nesticidae	79	16	44	12
Carabids	Omnivorous	A.g	*Amara gigantus*	2	0	0	1
		A.p	*Amara plebeja*	1	3	2	0
		D.g	*Dyschirius glypturus*	1	8	0	1
		H.c	*Harpalus cslceatus*	0	1	0	1
		H.sim	*Harpalus simplicidens*	25	0	1	0
		H.sin	*Harpalus sinicus*	0	0	0	0
		S.t	*Scarites terricola*	13	1	0	0
		T.g	*Tachys gradatus*	0	22	0	1
		T.n	*Tachys nanus*	12	13	2	4
	Carnivorous	C.l	*Calosoma lugens*	8	11	12	8
		C.j	*Chlaenius junceus*	4	1	0	0
		C.m	*Chlaenius micans*	27	17	8	5
		C.p	*Chlaenius praefactus*	1	1	0	0
		C.v	*Chlaenius variicornis*	0	1	0	0
		D.h	*Dolichus halensis*	12	3	2	0
		H.p	*Harpalus pallidipennis*	0	14	1	0
		L.c	*Lachnolebia cribricollis*	0	2	0	0
		P.d	*Panagaeus davidi*	1	0	0	0
		P.o	*Pheropsophus occiptalis*	7	7	0	3

**Table 2:** ANOSIM of spider and carabid community structures.

Comparisons	Spiders		Carabids	
	2021	2022	2021	2022
2m-T vs. 4m-T	0.0291	0.2811	0.0274	0.0579
2m-T vs. 6m-T	0.03	0.1134	0.0291	0.0868
4m-T vs. 6m-T	0.0327	0.5141	0.201	0.0327
2m-T vs. 2m-C	0.0302	0.0319	0.1411	0.7717
4m-T vs. 4m-C	0.0306	0.0291	0.028	0.0266
6m-T vs. 6m-C	0.0294	0.0278	0.0549	0.0294
R	0.6663	0.6751	0.2569	0.3125
*p*	0.001	0.001	0.001	0.001

The *p*-values from pairwise comparisons in the ANOSIM analysis were listed in row 1–6; the R-values and *p*-values for the overall group comparison were listed in row 7–8.

**Table 3:** Relationship between the active densities of spiders and carabids and the distance from the strip edge.

Groups	Years	Widths	Spearman Correlation Coefficients		ANOVA		
			r	*p*	F	df	*p*
Spiders	2021	2 m	−0.20	0.331	5.97	23	0.002
		4 m	−0.70	<0.001	7.45	23	<0.001
		6 m	−0.73	<0.001	4.86	23	0.006
	2022	2 m	−0.43	0.034	5.34	23	0.003
		4 m	−0.72	<0.001	16.02	23	<0.001
		6 m	−0.73	<0.001	62.64	23	<0.001
Carabids	2021	2 m	0.21	0.305	2.51	23	0.068
		4 m	−0.60	0.002	7.03	23	<0.001
		6 m	−0.36	0.78	0.94	23	0.479
	2022	2 m	0.25	0.226	1.71	23	0.183
		4 m	−0.79	<0.001	4.29	23	0.009
		6 m	−0.86	<0.001	19.17	23	<0.001

**Table 4:** Vegetation community composition within floral strips.

Years	Families	Species	Importance Values (%)
2021	Amaranthaceae	*Amaranthus blitoides* S. Watson	3.07
	Asteraceae	*Cichorium intybus* L.	18.08
		*Cirsium arvense* var. *integrifolium Wimm. & Grab*.	6.31
		*Cosmos bipinnatus* Cav.	2.1
		*Helianthus tuberosus* L.	1.26
	Campanulaceae	*Lobelia nummularia* Lam.	0.84
	Chenopodiaceae	*Oxybasis glauca* (L.) S. Fuentes, Uotila & Borsch	3.42
	Convolvulaceae	*Ipomoea nil* (L.) Roth	0.9
	Cucurbitaceae	*Acalypha australis* L.	6.4
	Leguminosae	*Medicago sativa* L.	10.31
		*Trifolium pratense* L.	2.13
		*Trifolium repens* L.	4.1
		*Vicia sepium* L.	1.81
	Malvaceae	*Abutilon theophrasti* Medicus	2.1
		*Hibiscus trionum* L.	4.26
	Moraceae	*Humulus scandens* (Lour.) Merr.	2.65
	Poaceae	*Digitaria sanguinalis* (L) Scop.	12.57
		*Lolium perenne* L.	0.59
		*Phragmites australis* (Cav.) Trin. ex Steud	2.07
		*Setaria viridis* (L.) P. Beauv.	13.22
	Solanaceae	*Alkekengi officinarum* Moench	1.81
2022	Asteraceae	*Artemisia caruifolia* Buch.-Ham. ex Roxb.	1.20
		*Aster indicus* L.	2.35
		*Cichorium intybus* L.	18.86
		*Cirsium arvense* var. *integrifolium* Wimm. & Grab.	10.88
		*Sonchus oleraceus* L.	6.47
	Leguminosae	*Medicago sativa* L.	17.75
		*Trifolium pratense* L.	1.82
		*Trifolium repens* L.	2.24
	Malvaceae	*Abutilon theophrasti* Medicus	1.72
	Moraceae	*Humulus scandens* (Lour.) Merr.	3.59
	Poaceae	*Lolium perenne* L.	10.33
		*Setaria viridis* L.	20.86
	Rubiaceae	*Galium spurium* L.	1.93

**Table 5:** Monte Carlo significance test of environmental factors to the RDA models.

Groups	Years	Environmental Factors	Explains (%)	Contributions (%)	F	*p*
Spiders	2021	Strip width	50.0	62.7	10.0	0.006
		Coverage	18.9	23.7	5.5	0.008
		Abundance	4.8	6.0	1.5	0.248
		Richness	2.5	3.1	0.7	0.55
		Shannon Diversity Index	2.0	2.5	0.5	0.67
		Pielou’s Evenness Index	1.6	2.0	0.4	0.766
	2022	Strip width	31.7	49.7	4.6	0.06
		Coverage	9.3	14.6	1.4	0.254
		Abundance	9.0	14.2	1.5	0.264
		Richness	8.8	13.8	1.5	0.25
		Shannon Diversity Index	2.6	4.1	0.4	0.64
		Pielou’s Evenness Index	2.4	3.7	0.3	0.632
Carabids	2021	Strip width	14.8	24.9	2.2	0.076
		Coverage	23.8	40.0	3.1	0.026
		Abundance	8.8	14.8	1.3	0.28
		Richness	1.3	2.2	0.2	0.974
		Shannon Diversity Index	6.7	11.2	0.8	0.526
		Pielou’s Evenness Index	4.1	7.0	0.6	0.666
	2022	Strip width	19.2	27.6	2.4	0.04
		Coverage	12.8	18.4	1.7	0.172
		Abundance	12.7	18.3	1.8	0.112
		Richness	9.1	13.1	1.4	0.236
		Shannon Diversity Index	3.5	5.1	0.5	0.82
		Pielou’s Evenness Index	12.2	17.5	2	0.116

## Data Availability

Data will be made available on request.
